# Cardiovascular safety pharmacology: beyond arrhythmic risk assessment

**DOI:** 10.3389/fcvm.2025.1623644

**Published:** 2025-12-09

**Authors:** Raafat Fares, Pascal Champéroux

**Affiliations:** ERBC France, Chemin de Montifault, Baugy, France

**Keywords:** autonomic nervous system, cardiac electrophysiology, cardiovascular pharmacology, drug safety, hemodynamic, cardiovascular risk assessment, safety pharmacology

## Abstract

Cardiovascular safety pharmacology arose from the need to assess certain forms of drug induced functional cardiotoxicity in toxicology within a regulatory framework. Cardiotoxic effects resulting from direct or indirect pharmacological effects are difficult to apprehend in conventional toxicology studies, whether electrophysiological and/or hemodynamic. The reflex regulatory systems of the cardiovascular system, and in particular the autonomic nervous system, interfere with and very often minimize the functional impact of these pharmacological effects, which are sometimes only visible over a very short time window. Modeling approaches now make it possible to assess key hemodynamic parameters, going beyond blood pressure alone. Non-clinical cardiovascular safety pharmacology must continue to evolve toward a comprehensive framework for arrhythmic risk, in order to improve its translational relevance to humans and better bridge non-clinical QT prolongation data with clinical risk assessment. It also needs to integrate concepts from clinical research, such as Coumel's triangle, autonomic conflict or hidden cardiotoxicity. The ultimate goal of cardiovascular safety pharmacology should extend beyond protecting participants in clinical trials. It should broaden its scope to include patient subpopulations with underlying cardiovascular disease, who are often the most vulnerable to functional cardiotoxic effects. Twenty-five years after their initial publication, the safety pharmacology guidelines are currently undergoing revision. This review aims to foster a more balanced and comprehensive approach to cardiovascular safety pharmacology, beyond arrhythmic risk.

## 1 Introduction

**Figure 1:**
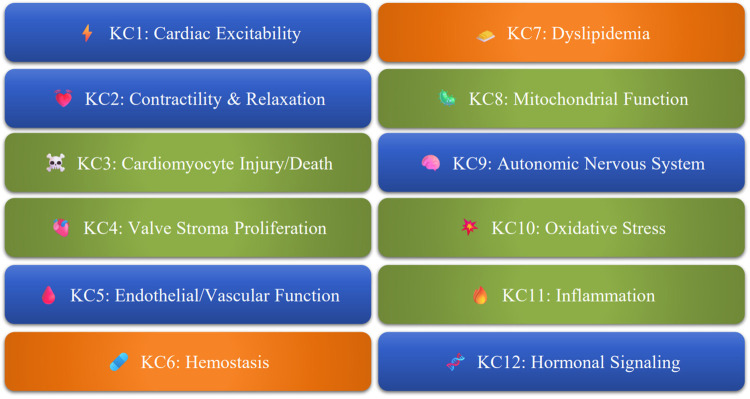
The 12 Key characteristics (KCs) of cardiotoxicity. KCs are grouped by their mechanistic impact on the cardiovascular system. Figure created by the authors based on (2). KCs are classified according to their susceptibility to functional cardiotoxicity: high (blue), mid (orange), low (green).

**Figure 2:**
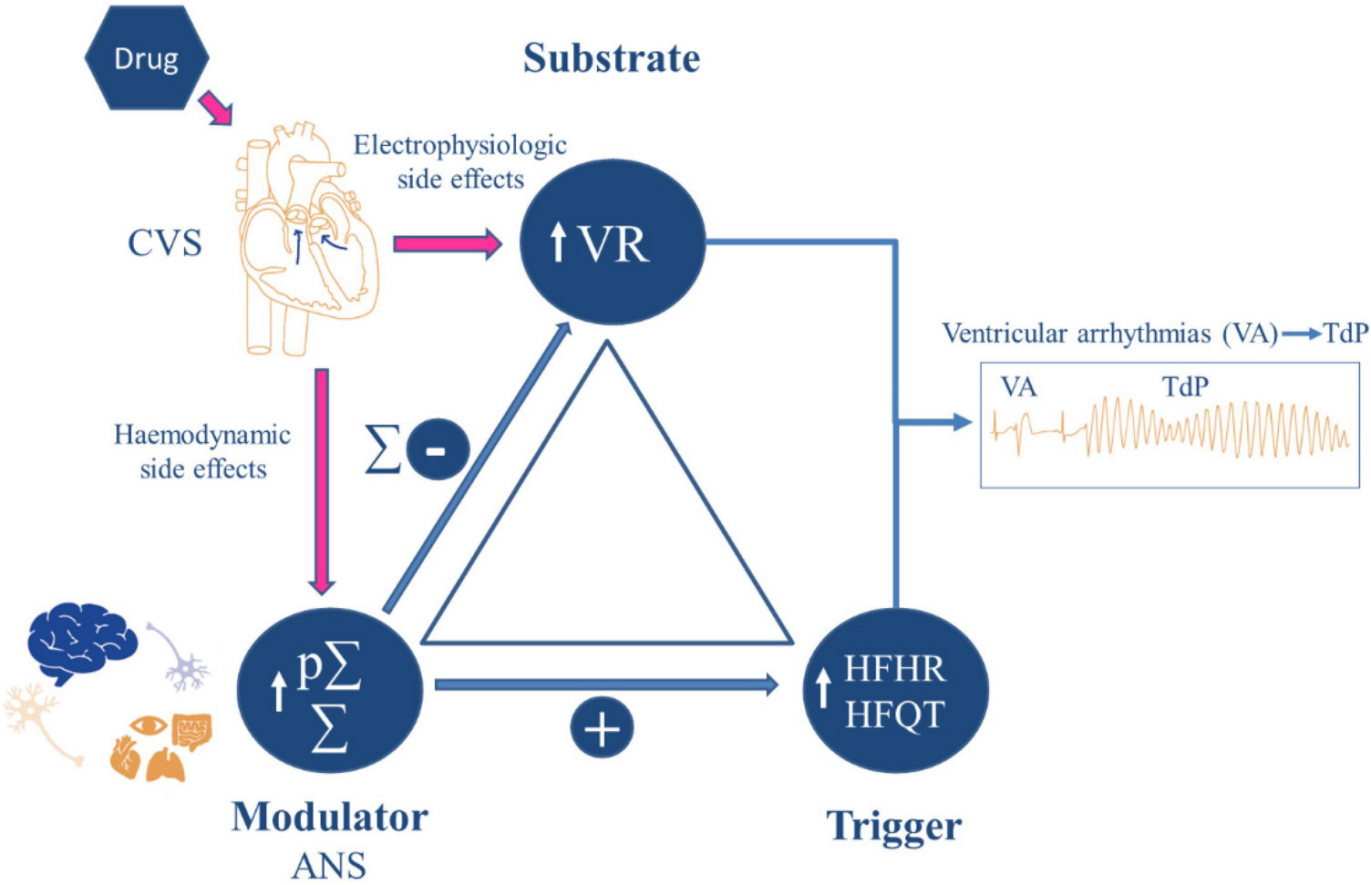
Coumel's triangle applied to hERG channel-blocking torsadogenic drugs. According to Coumel's triangle concept, ventricular arrhythmias and torsades de pointes (TdP) induced by hERG channel-blocking torsadogenic drugs may require: (1) a substrate, the prolongation of ventricular repolarization (VR); (2) a modulator, the autonomic nervous system (ANS), which is activated by the hemodynamic side effects of these drugs. It is worth noting that the sympathetic component of HF oscillations can shorten ventricular repolarization (VR) and conceal QTc interval prolongation; (3) a trigger, the increase in HFHR and HFQT oscillations induced by sympathetic-vagal coactivation in response to the hemodynamic effects. *Σ*: Sympathetic nervous system, p*Σ*: Parasympathetic nervous system. Reprinted with permission from (63), licensed under CC BY 4.0.

Cardiovascular safety pharmacology is a major component of safety pharmacology. The concept of safety pharmacology was established with the ICH S7A guideline (1). Safety pharmacology is now part of toxicology and focuses on functional toxicity mechanisms that are not necessarily well evaluated in regulatory toxicology. Safety pharmacology focuses on a limited set of vital functions i.e., cardiovascular function, respiratory function, and the central nervous system, referred to as the “Core Battery.” By prioritizing these vital functions and clinical trial safety, the guideline highlighted functional toxicities of pharmacological origin that pose significant risks to clinical trial participants. These functional toxicities typically arise from reversible and selective interactions, such as blocking or stimulating pharmacological targets like membrane or nuclear receptors, or enzymes with functional roles in the body. Small-molecule drugs are most commonly associated with this type of functional toxicity. However, despite their higher target specificity, gene therapy-based drugs, monoclonal antibodies, and microRNAs can also cause functional toxicity in addition to direct lesions, either directly related to their therapeutic targets or through off-target interactions. In total, 12 key characteristics (KCs) have been proposed to cover all identified mechanisms of cardiotoxicity [Figure 1, (2)]. The pharmacological mechanisms of cardiotoxicity that directly affect the primary functions of the cardiovascular system can be grouped into five categories, each with common key characteristics. These mechanisms of functional toxicity make reference mainly to Cardiac Excitability (KC1), Cardiac Contractility and Relaxation (KC2), Endothelial and Vascular Function (KC5), Autonomic Nervous System Activity (KC9), and Hormonal Signaling Pathways (KC12). Cardiovascular safety pharmacology initially focused on a major risk belonging to the KC1 category relating to cardiac excitability. This risk captured the attention of regulators and safety pharmacologists: the risk of torsades de pointes. These ventricular arrhythmias can be induced by certain cardiovascular drugs, including, paradoxically, antiarrhythmic medications. Non-cardiovascular drugs can also cause this type of arrhythmia, which may degenerate into ventricular fibrillation, potentially leading to sudden death. This risk is assessed by evaluating the potential for drugs to prolong ventricular repolarization, as this electrophysiological effect is known to be associated with an increased likelihood of arrhythmias (3). In practice, cardiovascular safety pharmacology is primarily limited to two regulatory studies, mostly conducted on small chemical molecules: the *in vitro* assessment of effects on the hERG channel and the *in vivo* assessment of cardiovascular effects, including QT interval assessment via telemetry. In theory, the stand-alone safety pharmacology studies using telemetry can be replaced by safety pharmacology endpoints integrated into regulatory toxicology studies involving repeated doses, even for small molecules. For medicines covered by the ICH S6 guideline (4) such as monoclonal antibodies and large molecules, pharmacological endpoints are integrated into toxicology studies. Other specific guidelines also follow this strategy of integrating safety pharmacology into toxicology studies, such as those for vaccines (5) and anticancer agents (6). These safety pharmacology endpoints primarily involve electrocardiogram (ECG) and blood pressure recordings, which are incorporated into repeated-dose toxicology studies using non-invasive telemetry (e.g., jacket telemetry for ECG) or oscillometry for blood pressure measurements. The regulatory focus on the arrhythmic risk has somewhat overshadowed other cardiovascular risks related to pharmacological effects. The effects of drugs on blood pressure must also be a major concern for safety pharmacologists and toxicologists, particularly following the 2022 publication of a draft FDA guideline on evaluating pressor effects in clinical trials (7). Cardiovascular safety pharmacology is set to evolve in regulatory terms through a revision of the ICH S7A and S7B guidelines. It must also continue to innovate in order to be increasingly predictive in the early stages of drug discovery and early safety. This review aims to foster a more balanced and comprehensive approach to cardiovascular safety pharmacology, beyond arrhythmic risk. This review focuses on essential topics that may be overlooked, underestimated, neglected, or rarely questioned.

## 2 Hemodynamic risk: looking beyond blood pressure

### 2.1 The autonomic nervous system: the overlooked player in safety pharmacology

Even minor changes, such as a few mmHg increase in blood pressure or a few beats per minute in heart rate, can lead to medium- or long-term adverse consequences, particularly in at-risk populations. For this reason, the FDA draft guideline requires that the upper bound of the 95% confidence interval for the mean systolic blood pressure (SBP) change to remain below 3 mmHg for clinical trials evaluating drugs intended for chronic use (7). This requirement is based on the ASCVD (Atherosclerotic Cardiovascular Disease) model, which predicts an increased risk of 0.5–1 cardiovascular event (e.g., stroke, myocardial infarction) per 1,000 patients over a 10-year period, beginning with a 3 mmHg increase in populations with intermediate to high cardiovascular risk (8). This 3 mmHg value represents the average blood pressure elevation measured over 24 h. To accurately assess this type of risk in cardiovascular safety pharmacology, it is essential to have a comprehensive understanding of the measured parameters, how they are regulated, and the underlying mechanisms involved.

When a drug is likely to influence blood pressure, the first system engaged to maintain stability is the baroreflex, also known as the high-pressure baroreflex (9). Understanding and assessing the effects of a drug on the autonomic nervous system is thus essential for predicting its impact on blood pressure. The baroreflex system is highly effective in compensating for various hemodynamic changes that could alter blood pressure. In healthy animals, these compensatory mechanisms can fully offset such changes, often without any visible fluctuations in blood pressure. However, in humans, the same hemodynamic disturbances may have severe effects, especially in certain populations or pathological contexts where the baroreflex is impaired and less efficient. This is observed, for example, in individuals with hypertension (10), diabetes (11), or in elderly populations (12), where baroreflex sensitivity tends to be reduced. Baroreflex activation is generally reflected in heart rate variations. Any change in heart rate should prompt consideration of a potential hemodynamic effect that could impact blood pressure, necessitating systematic investigation of its origin. However, if blood pressure remains unchanged, establishing a correlation between heart rate changes and the baroreflex is not possible. Numerous mechanisms other than the baroreflex can influence heart rate. A drug may directly affect heart rhythm or act on the central nervous system, which then relays its effects to the heart via the autonomic nervous system. Unfortunately, the effects of drugs on the autonomic nervous system are not commonly evaluated in safety pharmacology as a first-line approach.

The autonomic control is classically studied from vagally-driven oscillations called high-frequency (HF) oscillations because their frequency exceeds 0.1 Hz (105). In humans, spectral analysis of the power of HF rhythms in the heart rate or RR interval remains the gold standard for studying the autonomic nervous system and its dysfunctions in various pathophysiological situations or under the influence of drugs (13). This analysis is typically conducted over a 5-minute period while controlling the respiratory rhythm, often with the help of a metronome. The sympathetic system also exhibits rhythmic activity, but at much slower frequencies, below 0.1 Hz and sometimes as low as 0.05 Hz. These are referred to as low-frequency (LF) rhythms. However, this spectral analysis approach for heart rate variability (HRV) is not suitable for awake animals because it requires stable rhythms over several minutes. In ambulatory conditions, the autonomic nervous system's state can change within seconds, depending on activity, emotional state, or certain sleep phases, such as REM sleep (14). The HFAM model, which stands for “High Frequency Autonomic Modulation,” is an in silico model designed to assess the activity of both sympathetic and parasympathetic systems under ambulatory telemetry recording conditions. It allows quantification of autonomic nervous system activity over very short 10-second periods using simple algorithms based on a simple analysis of the magnitude of HF oscillations of beat-to-beat heart rate and RR interval (15). This is the only model capable of quantifying a particular and relatively unknown state of the autonomic nervous system that is characterized by a sympatho-vagal coactivation.

Another aspect is that the baroreflex response is extremely rapid, occurring within a single heartbeat when blood pressure exceeds or falls below its activation threshold. If the effect persists for several hours, other regulatory systems are engaged. These systems are numerous and activate more slowly, sometimes very slowly. They may be circulatory, tissue-based, or local, and can act as co-neurotransmitters or influence the central nervous system. Key systems include the renin-angiotensin system, bradykinin, arginine-vasopressin, adrenaline, neuropeptide Y, endothelin, nitric oxide (NO), and nitric oxide synthase. Over time, structural and morphological changes, remodeling phenomena, and modifications in neuronal densities or specific receptors in the cardiovascular or central systems (up- and down-regulation), may occur. These mechanisms collectively counteract the initial pharmacological hemodynamic effects of the drug to maintain stable blood pressure. Long-term structural and morphological changes resulting from hemodynamic effects may become evident and could be associated with hypertrophy, valvulopathy, and signs of myocardial necrosis (16, 17). However, in some cases, such changes may be subtle, undetectable, or observed only in a small subset of animals without clear dose-dependence in repeated-dose toxicity studies.

Incorporating blood pressure measurements into chronic toxicity studies often does not detect this type of hemodynamic pharmacological effect when it is compensated, particularly at intermediate or low doses. This limitation is not due to the lower precision of blood pressure measurements by oscillometry in toxicology compared to invasive telemetry (18) but rather to the exceptional effectiveness of blood pressure regulatory systems in young, healthy animals commonly used in regulatory toxicology studies. These systems are often capable of compensating for hemodynamic pharmacological effects in many cases. Similarly, heart rate variations are unlikely to be observed over the medium or long term. Reflex tachycardia or bradycardia typically only lasts for a few days, as the baroreflex quickly readjusts its activation thresholds, allowing other blood pressure regulatory systems to take over and compensate for the drug's hemodynamic effects (19). This phenomenon, known as “baroreflex resetting,” highlights the adaptability of these regulatory mechanisms. Given these regulatory dynamics and their time course, the best opportunity to detect the hemodynamic pharmacological effects of a drug is during the first administration. At this time, the drug's impact on blood pressure and/or the amplitude of the reflex response in heart rate variation is most likely to be evident. However, in toxicology studies, the first day is typically dedicated to toxicokinetic sampling, making it highly likely that the peak hemodynamic effect will be missed by the second day of administration. These remarks do not suggest that blood pressure measurements by oscillometry are without value in toxicology studies. Such measurements are essential for monitoring and assessing the general health status of animals and for identifying cases of decompensation resulting from significant hemodynamic effects. Repeated-dose toxicology studies using invasive telemetry at toxic doses are occasionally conducted when a drug causes unexplained mortality without apparent cardiovascular histopathology or prior clinical signs. These studies are valuable for diagnosing the cause of death, which is typically either arrhythmic (e.g., ventricular fibrillation) or hemodynamic (e.g., sudden cardiac function decompensation). Outside such cases of unexplained mortality, invasive telemetry in repeated-dose toxicology studies at toxic doses and durations longer than 1–2 weeks is unlikely to yield additional useful information about pharmacological hemodynamic mechanisms. A single-dose study or a short treatment duration of a few days is generally more effective for identifying these effects, given the role of blood pressure regulatory systems in compensating over short, medium, and long terms. The likelihood of observing a hemodynamic effect is considered to be low for drugs that fall outside the scope of the ICH S7A guideline, such as large molecules or monoclonal antibodies. In most cases, the cardiovascular effects of these biologics are evaluated in toxicology studies with the assumption that their drug profile has a low probability of interfering with the hERG channel or prolonging the QT interval (20). However, the risk of hemodynamic effects should not be overlooked. These effects could be better assessed in a cardiovascular safety pharmacology study using telemetry, even if only a single dose is tested. This option is proposed in the ICH S7A guideline. In the field of kinases and phosphatases, numerous intracellular signaling pathways and targets have been identified as potential sources of hemodynamic effects that could influence cardiac contractility, vascular resistance, or cause electrophysiological changes affecting ventricular repolarization (21). Additionally, correlations between morphological changes in the heart and vessels observed in repeated-dose toxicology studies and the effects on blood pressure and/or heart rate detected in single-dose cardiovascular safety pharmacology studies show that morphological changes are almost always linked to detectable changes in blood pressure or heart rate (22). In contrast, about 80% of molecules that affect blood pressure and/or heart rate do not cause cardiovascular histopathology. These findings reinforce the idea that, in most cases, compensatory reflex mechanisms minimize the long-term consequences of a drug's hemodynamic effects. The absence of morphological changes in toxicology studies does not exclude potential hemodynamic effects. Therefore, drugs, including biologics, whose effects on blood pressure are only evaluated in repeated-dose toxicology studies, are more likely to have undetected hemodynamic effects in the preclinical phase compared to small molecules. Table 1 highlights the key points regarding the importance of modeling autonomic nervous system activity.

**Table 1:** Key points regarding the importance of modeling autonomic nervous system (ANS) activity.

The importance of modeling ANS activity	
Key point	Purpose and importance
Regulation of cardiovascular stability	The ANS controls blood pressure and heart rate through parasympathetic and sympathetic pathways, crucial for maintaining hemodynamic balance and detecting drug-induced effects.
Baroreflex and adaptive mechanisms	Evaluating the baroreflex and its interaction with slower regulatory systems (e.g., renin-angiotensin, nitric oxide) helps understand both immediate and long-term cardiovascular adaptations.
Markers of autonomic activity	High-frequency (HF) oscillations (parasympathetic) and low-frequency (LF) rhythms (sympathetic) provide quantitative insights into autonomic responses and drug impacts.
Early detection of effects	Single-dose telemetry studies reveal initial, uncompensated drug effects on cardiovascular function, which may be masked in chronic studies by regulatory compensations.
Dynamic and versatile assessment	Tools like the HFAM model enable real-time, short-term evaluation of autonomic states, capturing transient parasympathetic and sympathetic activity in ambulatory conditions.

### 2.2 Modeling of key hemodynamic parameters

One often-overlooked aspect of blood pressure in safety pharmacology is the variation depending on the measurement site. The farther the measurement site is from the heart, the higher the systolic blood pressure tends to be (23). This phenomenon, known as systolic blood pressure amplification, occurs in peripheral arteries due to reflected waves from arterial territories that return to the left ventricle (24). This amplification can be significant. For example, in dogs, it ranges from 20 to 30 mmHg on average at the level of the abdominal aorta when using telemetry (25). The only exception to this is central aortic pressure in the ascending aorta, which is not subject to this amplification effect. Unfortunately, measuring blood pressure in the ascending aorta with telemetry presents technical challenges. In humans, blood pressure is typically measured in ambulatory conditions at peripheral arteries, such as the brachial or radial arteries. Algorithms based on transfer functions model the central aortic pulse wave from the peripheral artery pulse waves, allowing for unbiased blood pressure readings (26). Moving average-based algorithms (NPMA or N-point moving average) are also used to filter this amplification effect (27). This approach is simpler than transfer functions and provides reliable estimates of central aortic systolic pressure via telemetry. The amplification phenomenon varies between individuals, contributing to individual variability. It also depends on how drugs affect the vascular resistance in peripheral arteries. In some cases, this phenomenon can mask a drug's effects on systolic blood pressure (28). Therefore, evaluating a drug's impact on systolic blood pressure is more sensitive when using modeled central aortic pressure rather than measurements from peripheral arteries. Diastolic blood pressure is also affected, but to a lesser extent than systolic blood pressure. Modeling the pulse wave of central aortic pressure also enables the modeling of additional important hemodynamic parameters, including stroke volume. There are many models available, ranging from simple to sophisticated, initially grouped under the term “Pulse Contour Method.” The simplest models rely on the well-known principle that the area under the central aortic pulse wave curve is proportional to stroke volume during systole (29). The end of systole is estimated by the appearance of the dicrotic wave, which marks the closing of the sigmoid valve. Once stroke volume is modeled, it becomes possible to derive estimates of cardiac output and systemic vascular resistance. Access to these key hemodynamic parameters allows for a much more detailed evaluation of a drug's hemodynamic effects and an estimation of its safety margin. As previously explained, blood pressure is the last parameter to change in response to a drug's hemodynamic effect. When blood pressure is altered, it indicates that the reflex regulatory systems have failed to compensate for the drug's direct hemodynamic effects. Conversely, the absence of blood pressure variation does not mean that the drug has no hemodynamic effect. The case of verapamil perfectly illustrates the value of in silico modeling of these key hemodynamic parameters (25). At a relatively high dose administered in a single dose, this L-type calcium channel blocker has no effect on blood pressure but induces a moderate increase in heart rate, suggesting baroreflex activation. Confirmation of the hemodynamic effect is provided by modeling systemic vascular resistance, which is greatly reduced as expected with a vasodilator like verapamil. This mechanism explains the baroreflex activation, which functions to maintain stable blood pressure. In the case of verapamil, the baroreflex completely compensates for the vasodilatory effects, primarily through an increase in cardiac output resulting from sympathetic activation, as confirmed by the HFAM model. Without modeling the activity of the autonomic nervous system and the underlying hemodynamic parameters, the functional consequences on blood pressure of verapamil would likely have been under-evaluated in standard stand-alone safety pharmacology study. In the absence of visible effects on blood pressure, this profile might initially have seemed not to warrant further investigation. Modeling key parameters such as stroke volume, cardiac output, and systemic vascular resistance from telemetry-based blood pressure signals also allows for the detection of hemodynamic effects on a vascular compartment that is rarely considered in cardiovascular safety pharmacology: the venous compartment. The effects of drugs on this compartment are not systematically studied and are difficult to assess because venous pressure is very low. This compartment functions as a reservoir of blood volume, distributing blood between the venous and arterial compartments. It is innervated by the sympathetic nervous system, with alpha-1 adrenergic receptors playing a significant role in venous vascular tone (30). Their stimulation causes venous vasoconstriction. Venous blood flow into the right atrium, also known as venous return, activates reflex pathways involving volume receptors and baroreceptors located in the four cardiac chambers. These reflexes lead to cardio-inhibitory effects, causing vagal bradycardia and decreased sympathetic activity, resulting in peripheral vasodilation and hypotension (31). The Bezold-Jarisch reflex, which can also be activated via chemoreceptors, is one such cardio-inhibitory reflex. These reflexes are triggered in cases of hemorrhage, hypovolemia, or changes in venous return. It is important to consider these reflexes, as they may play a crucial role in the onset of drug-induced syncope (32). A typical example is molecules with antagonist properties at alpha-1 adrenergic receptors, which block both arterial and venous alpha-1 adrenergic receptors. By blocking the venous alpha-1 adrenergic receptors, they cause venous vasodilation, resulting in a redistribution of blood volume into the venous compartment, accompanied by a decrease in venous return. This redistribution of blood volume, along with reduced venous return, is responsible for vasovagal syncope and orthostatic hypotension, which are well-known with alpha-1 adrenergic receptor antagonists such as prazosin, thioridazine, or chlorpromazine (33). Through in silico modeling of stroke volume, it is possible to detect this type of deleterious hemodynamic effect. Indeed, these alpha-1 blockers all cause a decrease in stroke volume and cardiac output. These effects were described in awake animals using sonomicrometry techniques (34). In the case of alpha-1 blockers, this reduction in cardiac output is due to a decrease in venous return following the blockade of sympathetic control of venous compartments. In a closed circuit, the output flow is equal to the input flow. If the input flow is reduced, the “output” cardiac flow must necessarily be reduced. The effects of alpha-1 blockers on cardiac output and stroke volume are very characteristic in dogs, as they last for a long time, even at low doses (35). These effects are associated with a characteristic sympathetic-vagal coactivation, which is reflected by an increase in S2 oscillations in the HFAM model, becoming the predominant frequency. In the case of alpha-1 blockers, the baroreflex is deactivated by the decrease in blood pressure. This is visible on systolic blood pressure and leads to sympathetic activation. At the same time, the decrease in venous return activates cardio-inhibitory reflexes, resulting in vagal activation and significant slowing of the heart rate. The simultaneous activation of both branches of the autonomic nervous system (sympathetic and parasympathetic) during episodes of orthostatic hypotension has been confirmed by direct recording of the electrical activity of the sympathetic system and the vagus nerve at the cardiac level (36). In silico modeling of stroke volume also provides an advantageous alternative to measuring left ventricular pressure to detect negative inotropic effects that may lead to cardiac depression. Indeed, such deleterious effects can be detected through a decrease in cardiac output, as seen with high doses of quinidine or moxifloxacin (25). Cardiac output is an overall index of cardiac ejection performance. It is the product of stroke volume and heart rate, and it varies based on oxygen demand. The main determinant is the increase in heart rate compared to the increase in the strength of contractions, which also contributes to increasing stroke volume and thus cardiac output. There is a relationship between stroke volume and heart rate, ensuring that cardiac output remains generally stable regardless of heart rate variations, as long as oxygen demands remain constant. Therefore, a pharmacological agent like atropine can cause tachycardia exceeding +100 bpm without increasing cardiac output (35). Conversely, cardiac contractility, as estimated by the maximum rate of pressure rise in the left ventricle during the isovolumetric phase, where the aortic valve is closed (dLVP/dtmax), depends on the left ventricular end-diastolic volume, which in turn depends on filling time, itself dependent on heart rate. In other words, dLVP/dtmax is dependent on heart rate, just like stroke volume. Interpreting a drug's effects on cardiac contractility and stroke volume must consider their relationship with heart rate (37). This is directly measurable via cardiac output, as this parameter should not vary with changes in heart rate alone when oxygen demand remains constant. In the case of increased cardiac output, any modification of sympathetic system activity should also be considered. Indeed, an increase in sympathetic system activity, whether reflexive or otherwise, leads to an increase in cardiac output due to the positive inotropic effects of norepinephrine released at sympathetic nerve terminals innervating the left ventricular myocardium. These effects of norepinephrine are due to β1-adrenergic receptor stimulation. Therefore, one should not conclude a direct positive inotropic effect of the drug when cardiac output increases alongside concurrent sympathetic activation. The only way to confirm a positive or negative inotropic effect is to examine the effect directly on isolated heart models, isolated cardiac tissues, or human cardiomyocytes derived from stem cells, stimulated at a constant frequency. This latter model has the advantage of avoiding animal use and working directly with human-derived cells. Table 2 highlights the critical role of modeling key hemodynamic parameters.

**Table 2:** Highlights on the critical role of modeling key hemodynamic parameters.

The critical role of modeling key hemodynamic parameters	
Key point	Purpose and importance
Central aortic pressure vs. Peripheral measurements	Central aortic pressure modeling avoids any bias due to systolic amplification seen in peripheral arteries, providing a more accurate assessment of drug effects.
Improved sensitivity to drug effects	Modeling of hemodynamic parameters like stroke volume (SV), cardiac output, and systemic vascular resistance offer deeper insights into drug safety
Cardiac output and contractility dynamics	Cardiac output integrates stroke volume and heart rate changes, reflecting drug-induced inotropic and chronotropic effects while considering oxygen demand and sympathetic activation.
Venous compartment and autonomic responses	In silico modeling of hemodynamic and autonomic parameters allows detection of effects on the venous compartment and complex autonomic responses like sympatho-vagal coactivation, which are critical for evaluating syncope and orthostatic hypotension risks.
Reflex and compensatory mechanisms	Monitoring blood pressure combined with modeling of hemodynamic and autonomic parameters help to identify compensatory mechanisms like baroreflex activation or deactivation, which can mask direct drug effects.

## 3 Cardiac electrophysiological risks: looking beyond the hERG channel

### 3.1 Towards a comprehensive framework for arrhythmic risk

The risk of torsades de pointes (TdP) caused by certain non-cardiovascular drugs was a key factor in establishing the ICH S7A and S7B guidelines, which are foundational to safety pharmacology. While these guidelines represent significant progress, preclinical evaluation strategies for assessing QT interval prolongation, an indicator of TdP risk, have proven insufficiently predictive for humans (38). This complexity provides an opportunity to deepen our understanding of TdP risk and improve methods for evaluating cardiac electrophysiological risks. The role of the IKr current generated for the hERG channel was quickly highlighted because this current is blocked by many drugs known to induce TdPs. The outward potassium IKr current is involved in ventricular action potential repolarization. It is mediated by the hERG channel in humans (39). Blocking this current delays repolarization, leading to prolonged ventricular repolarization and QT interval prolongation. As early as 2003, a retrospective analysis of available data in the literature on a large number of torsadogenic drugs or those labeled with a risk of QT interval prolongation revealed a poor correlation between the maximum therapeutic concentration and the IC50 value for the hERG channel (Cmax/IC50 ratio). As a precaution, the authors recommended maintaining a safety margin of 30 times the Cmax to avoid the risk of QT interval prolongation (40). In 2013, an initiative was launched bringing together regulatory authorities (FDA, Europe, Canada, Japan), academics, and industry to improve the predictability of non-clinical translational studies to clinical outcomes in terms of arrhythmic risk. This initiative is called CiPA, which stands for “Comprehensive *in vitro* Proarrhythmia Assay” (41). Various approaches were evaluated, such as the patch-clamp technique applied to seven of the main ion currents involved in cardiac action potentials: Ikr (hERG), IKs, ICaL, fast INa, late INa, Ito1, and IK1. The results obtained were used to create in silico models based primarily on modified versions of the O'Hara–Rudy model (42). These models aimed to predict the effects of action potential prolongation and the occurrence of early afterdepolarizations (EADs). The advantage of these *in vitro* models used in patch-clamp is that they allow the study of cloned human versions of ion channels. However, it should be emphasized that these channels are expressed in cells that are not cardiac cells, such as HEK 293 (Human Embryonic Kidney) cells. As a result, signaling pathways, including the various types of kinases that regulate ion channels, are necessarily different. These are factors that can contribute to discrepancies between the *in vitro* model and actual *in vivo* clinical reality in terms of effective concentrations, as reported in the 2003 retrospective analysis for the hERG channel (40). Moreover, evaluations conducted within the CiPA framework revealed unresolved intra- and inter-laboratory variability issues with automated patch-clamp techniques (43). Automated patch-clamp models were used for high-throughput screening of molecules in the early phases of R&D to identify the potential of a molecule to block specific ion channels. Action potential models using microelectrodes on cardiomyocytes derived from human stem cells were also evaluated within the CiPA framework. These studies showed that the gene expression of sodium, calcium, and hERG channels differed in stem cell-derived cardiomyocytes compared to adult human cardiomyocytes and exhibited some degree of immaturity (44). However, they are closer to *in vivo* models than action potential models performed on animal cardiac cells. The fact that these models exhibit spontaneous beating also raises the issue of correcting repolarization duration based on the beating frequency, following the same principle as QT interval correction by heart rate in *in vivo* models. Overall, validation results using known reference drugs showed satisfactory correlations with clinical outcomes. Like patch-clamp techniques, inter-laboratory validation studies highlighted the need for harmonization and standardization of methods and protocols used for studies on human stem cell-derived cardiomyocytes (45). The CIPA initiative allowed the repositioning of arrhythmic risk assessment within a more comprehensive and innovative framework. After initially focusing on IKr and IKs currents, the role of the “late INa” sodium current in ventricular repolarization must also be highlighted from a pharmacological safety perspective. This current is involved in the initial phase of action potential repolarization and contributes to prolonging the duration of repolarization. Blockade of this current by ranolazine confers a low arrhythmic risk profile despite its hERG channel blocking properties (46). Conversely, enhancement of this current prolongs the duration of the cardiac action potential. This mechanism of increasing the “late INa” current has been highlighted with several torsadogenic drugs, including dofetilide and sotalol (47). These two hERG channel blockers strongly prolong the duration of ventricular repolarization. This additional mechanism of enhancing the “late INa” current explains why these molecules produce some of the most significant QT interval prolongations induced by hERG channel blockers (48). The effects of these drugs on the “late INa” current, as well as on the hERG channel, are often “reverse-use” dependent and thus inversely dependent on heart rate (49). This results in an increased risk of significant QT interval prolongation under low heart rate conditions. The risk of torsades de pointes thus becomes particularly important under conditions of bradycardia or a slowed heart rate with this type of hERG channel blockers. The “late INa” sodium current is also enhanced by certain drugs with a multichannel profile, such as thioridazine (47) and terfenadine (50), which are also two molecules classified as having a high torsadogenic risk. The effects of these hERG channel blockers on the “late INa” sodium current are due to the inhibition of the phosphoinositide 3-kinase (PI3 K) pathway. An enhancement of the “late INa” current has also been observed with oncology drugs that inhibit the PI3 K pathway, such as nilotinib (50), leading to a decrease in intracellular phosphatidylinositol 3,4,5-trisphosphate (PIP3). Inhibition of this ion channel phosphorylation pathway results in the enhancement of the “late INa” current and a reduction of the IKr, IKs, ICaL, and “fast INa” currents. Despite the shortening in action potential duration due to the reduction in ICaL and “fast INa” currents, the overall result of all these effects is a prolongation of the repolarization duration (51). These effects can also lead to an increase in intracellular calcium and cause calcium overload, which is also arrhythmogenic (52). When associated with hERG channel blocking properties, this effect on the “late INa” current, combined with hERG channel blockade, appears to be a key factor in characterizing a high torsadogenic risk profile. This profile includes several drugs classified as high torsadogenic risk, such as dofetilide, sotalol, terfenadine, and thioridazine. It should be noted that studying the effects on this pathway of “late INa” current enhancement requires exposure times of several hours (>2 h) to reveal the effects on the “late INa” current due to transcriptional inhibition mechanisms, as observed with certain tyrosine kinase inhibitors. Some drugs cause a decrease in the expression of the hERG channel, known as “hERG trafficking,” such as pentamidine or probucol, leading to prolonged ventricular repolarization and QT interval, which also takes some time to develop and become visible (53).

### 3.2 Bridging non-clinical QT prolongation data to clinical risk assessment

The issue of statistical sensitivity is one of the topics addressed in the “Good Practices Q&A ICH S7B” (54). A lack of statistical sensitivity can indeed contribute to the high rate of false negatives in *in-vivo* studies compared to clinical studies. In fact, the number of animals included in non-clinical *in-vivo* studies is typically very low in order to minimize animal use. The standard experimental plan typically includes 4 treated animals in a crossover design using a 3-dose Latin square and a vehicle or placebo session (55). However, statistical sensitivity depends on the sample size of the experimental design. In contrast, clinical TQT studies are conducted with several dozen subjects to reach the sensitivity thresholds defined in the ICH E14 guidelines. The objective in clinical studies is to detect a QT interval prolongation of 10 ms, or even 5 ms, in the absence of or with a very narrow safety margin. It is important to note that the statistical approach differs in TQT studies compared to non-clinical *in vivo* studies. In clinical studies, the statistical analysis aims to exclude the risk of a QT interval increase greater than 10 ms. This risk is considered low when the upper limit of the 90% confidence interval for a one-sided comparison does not exceed the 10 ms threshold (56). Similarly, the sample size is calculated to demonstrate an increase of at least 5 ms, with moxifloxacin systematically included as a comparator in TQT studies. In animal studies, the presence of a positive control like moxifloxacin is not required by regulations in each study. The statistical approach in non-clinical studies differs, as it would require a large number of animals to reduce the standard deviation. These sample sizes would likely be similar to those in clinical studies, which is inconceivable for animal studies. The methodology used in non-clinical *in vivo* studies is based on the concepts of Least Significant Difference (LSD) and Minimum Detectable Difference (MDD) (55). To comply with the “Good Practices Q&A ICH S7B”, it is essential to demonstrate the ability of studies to detect effects of the same magnitude as the thresholds defined in clinical studies, namely 10 ms, or even 5 ms depending on the exposure levels. In other words, the goal is not to exclude the risk of a QT prolongation greater than 10 ms, but to be able to demonstrate QT prolongation in comparison to the control group with a sensitivity at least equal to the threshold defined for clinical studies, i.e., 10 ms or 5 ms depending on the safety margins. These sensitivity and statistical power constraints are clearly necessary for better translation of non-clinical data to the clinic. They are especially crucial because the thresholds defined in clinical settings are relatively low. Indeed, 5 and 10 ms represent 1% and 2% increases in the QT interval in humans, respectively. In practice, it is possible to achieve these sensitivity thresholds with small sample sizes in non-clinical studies by carefully controlling the factors that influence QT interval variability. The quality of the electrocardiograms must be perfect. The non-invasive “jacket telemetry” technique, commonly used in toxicology studies, does not allow for the level of quality achieved with invasive telemetry, even though it can be considered very good quality overall, especially for dogs. Epicardial electrode placement allows for the required quality level to be achieved in cynomolgus macaques, for which subcutaneous ECG placement is not sufficient (57). The technique of implanting a floating electrode in the cava vein near the heart enables nearly the same level of quality as epicardial placement (58), thus avoiding the need for major thoracic surgery. The quality of ECGs must be perfect to allow for automated beat-by-beat analysis, which in turn enables the determination of an average QT interval value over a large number of beats. The probabilistic method recommends a minimum of 250 beats in dogs and 500 beats in cynomolgus macaques to minimize the influence of the hysteresis phenomenon on the variability of the QT interval (57). This phenomenon can be observed during changes in heart rate, such as during physical exercise. It results in a delay in the adaptation of the QT interval duration when there is an increase or decrease in heart rate. The delay in adaptation is not systematic and generally does not exceed one minute. The thresholds of 250 beats for the dog or 500 beats for the macaque correspond to the calculation of an average QT interval value over about 5 min, thus smoothing out the hysteresis phenomenon. When the kinetics of the effects allow, as is the case for an orally administered drug, the best option is to calculate an average value per hour over the entire circadian cycle, especially since the effects of drugs on the QT interval resulting from hERG channel blockade are always lasting and are observed for at least several hours.

Another technical aspect that significantly influences the translational value of nonclinical cardiovascular safety pharmacology studies is QT interval correction. The risk of QT interval prolongation is always assessed by correcting the QT interval for heart rate or the RR interval due to the relationship between the QT duration and heart rate. The goal of these mathematical correction methods is to make the QT interval independent of any heart rate variation that a drug could induce. Older correction methods, such as the Bazett, Fridericia, and Van de Water formulas, do not achieve this independence (59). These methods result in either underestimations or overestimations of the drug effects on the corrected QT (QTc) values, depending on the species and the direction of heart rate changes. More recent correction methods involve linearizing the QT/RR or QT/HR relationship through linear regression to calculate the slope of the relationship, then applying a correction factor to the slope so that the slope of the QTc/RR or QTc/HR relationship becomes zero. Thus, any variation in QTc will result solely from an effect on ventricular repolarization, without being influenced by any concomitant changes in heart rate. All correction methods based on linear regression calculate the slope of the QT/RR or QT/HR relationship over a typically untreated 24-hour period. The slope is calculated individually for each animal, as the relationships exhibit notable variability between animals. This approach is referred to as the individual correction method. In the case of the QT/RR relationship, the slope is calculated after applying a Log/Log transformation to both the QT interval and the RR interval (LogQT/LogRR). This Log/Log transformation aims to linearize the QT/RR relationship, which is not always linear across species (60). In dogs, in particular, the QT/RR relationship exhibits varying degrees of curvature depending on the animal. The QT interval decreases sharply for low RR values, and the QT/RR relationship shows a plateau for high RR values. In this species, the Log(QT)/Log(RR) transformation linearizes the relationship, but the linearization remains imperfect, with a slight curvature that can introduce residual variability when there are changes in heart rate. In contrast, the relationship between the QT interval and heart rate is highly linear and does not require any Log/Log transformation. It is therefore preferable to correct the QT interval using a slope calculated from the QT/HR relationship. It is also important to note that all individual QT correction methods use the slope calculated in the absence of treatment over a long period, typically 24 h, to correct the QT interval. These methods assume that the slope of the QT/RR or QT/HR relationship remains the same throughout the circadian cycle, both in the absence or presence of treatment with a drug that may modify the duration of ventricular repolarization. These assumptions are not always accurate and may lead to incorrect conclusions in certain cases. Indeed, it has been shown that certain torsadogenic drugs cause abnormal QT hysteresis boundary (61) and changes in QT/RR slopes. A method known as “One-step QTc” is based on the hypothesis that this slope may vary depending on the drug induced effects (62). The approach involves calculating the slope of the QT/RR or QT/HR relationship on an individual animal basis, for each hour of the day, and separately in the absence and presence of treatment. The slope of these relationships is determined hourly via linear regression using the mean QT interval values computed every minute. This method allow to achieve a very high level of statistical sensitivity, with least significant difference (LSD) values in the range of 2–3 ms at the individual level. The term “one-step” indicates that the slope calculation is directly integrated into the QT interval correction model. Unlike other individualized correction methods, this approach does not require a prior slope calculation under control conditions without treatment. This method proves particularly useful for detecting the effects of certain torsadogenic drugs with multichannel profiles, which induce limited effects on ventricular repolarization duration while also affecting heart rate. Effects on heart rate with torsadogenic drugs are common, generally moderate, and typically result in an increase in heart rate (63). In certain cases, such as with thioridazine, even traditional individual correction methods, such as the probabilistic method, fail to demonstrate any QTc prolongation (59). Conversely, the “one-step QTc” method, based on the QT/HR relationship, demonstrates its ability to reveal QTc prolongation because it accounts for changes in the slope of the QT/RR or QT/HR relationship during treatment (64). This lack of QTc interval prolongation with traditional individual correction methods has been termed “concealed QTc prolongation,” as the use of a beta-blocker to inhibit the sympathetic activation induced by these drugs reveals their ventricular repolarization prolonging properties (63).

### 3.3 The coumel triangle

The term “concealed LQT” was first introduced in clinical settings to describe the observation that approximately 25% of patients genotyped with congenital Long QT Syndrome (LQTS) exhibit normal QT and QTc interval values (65). There are several types of congenital Long QT Syndromes. The three most common are LQT1, LQT2, and LQT3 syndromes, which result from mutations in the KCNQ1, KCNH2 (hERG), and SCN5A genes, respectively (66). These mutations affect subunits of potassium and sodium ion channels, leading to a reduction in IKs currents (LQT1), IKr currents (LQT2), and a persistence of the late INa current (LQT3), respectively. These patients are among the populations most at risk of developing torsades de pointes. The risk of triggering torsades de pointes, and consequently sudden cardiac death, increases when these patients are exposed to a drug that could further prolong ventricular repolarization, which is already prolonged or altered due to a genetic mutation. According to Coumel's triangle theory (67), cardiac arrhythmias almost always require three components: a substrate, a trigger, and a modulator. Applied to the risk of torsades de pointes, the prolongation of repolarization serves as the substrate for triggering torsades de pointes. The pathological context and the drug itself are typically the two main contributors leading to reduced repolarization reserve (68). The primary modulator is often the autonomic nervous system. In the context of ventricular repolarization, the autonomic nervous system modulates the duration of repolarization through its effects on heart rate, cardiac conduction, and directly on ionic currents such as IKs and ICaL. Few molecules are truly arrhythmic and capable of triggering arrhythmias in animals or healthy subjects without any particular substrate, solely due to their electrophysiological properties. An example is Bay K 8644, a calcium channel agonist. This molecule increases the likelihood of calcium channel opening, causing an influx of calcium into the cell, leading to calcium overload responsible for early depolarization phenomena at the plateau of the action potential. Another example is digitalis, which triggers arrhythmias by inhibiting the Na/K pump, resulting in calcium overload. The M cells of the subendocardial myocardium are highly sensitive to these two arrhythmic mechanisms (69). In both examples, the direct arrhythmic effects are significant enough to be easily detectable in healthy animals, including in toxicology studies. Among the triggering factors, the sympathetic nervous system is one of the most common triggers of cardiac arrhythmias due to its effects on intracellular calcium load, cardiac conduction, and/or heart rate (70). The prolongation of ventricular repolarization favors calcium overload phenomena, which can lead to early afterdepolarizations (EADs), which in turn can trigger arrhythmias. The triggering of ventricular arrhythmias related to the prolongation of ventricular repolarization can also be due to reentry mechanisms within the myocardium, originating from the M cells of the subendocardial myocardium (71). This subpopulation of cardiomyocytes is particularly sensitive to mechanisms that prolong the repolarization of action potentials, such as the blockade of the hERG channel. The Purkinje fibers, which line and infiltrate the endocardium, have a similar profile to M cells and are also implicated in reentry mechanisms that trigger ventricular arrhythmias and torsades de pointes. These arrhythmic mechanisms due to reentry are caused by the transmural dispersion of action potential repolarization duration and refractory periods within different cell populations in the ventricular wall. The pathological context, such as myocardial ischemia, heart failure, diabetes, or long QT syndromes, also plays a crucial role in determining whether the arrhythmic potential of many drugs becomes apparent (72). These pathologies can be considered as contributing parts of the substrate necessary for the triggering of ventricular arrhythmias induced by torsadogenic drugs, as diabetes and heart failure lead to prolongation of ventricular repolarization, while myocardial ischemia promotes reentry circuits (73). In the absence of associated pathology, as seen in healthy dogs, few hERG channel blockers directly trigger ventricular arrhythmias, and none induce torsades de pointes. Among the hERG channel blocker torsadogenic agents, only dofetilide is truly arrhythmic, leading to a significant increase in the number of ventricular arrhythmias over 24 h. The inter-individual sensitivity to the arrhythmic effects of dofetilide remains highly significant. Some animals do not trigger any arrhythmias with dofetilide, even at relatively high doses. This molecule is also one of the few, along with astemizole, to induce torsades de pointes in cynomolgus monkeys (74) and marmosets (75). In dogs, several proarrhythmic drugs increase the frequency of spontaneous ventricular arrhythmias (63). To detect this type of very weakly arrhythmic profile in healthy animals, an automated arrhythmia detection system is needed, using ECGs with perfect signal quality, such as that obtained by the floating electrode technique placed in the cava vein (58). The algorithms must be capable of detecting arrhythmias with a resolution of 1–2 arrhythmias per 100,000 beats over 24 h. Indeed, most animals experience spontaneous arrhythmias, but their frequency over 24 h is very low (76). These data are very different from the arrhythmia prevalences published from short-duration external ECG recordings (77, 78), such as those used in repeated-dose toxicology studies. Attributing an increase in the frequency of spontaneous arrhythmias to a medication requires a comparison with a substantial database to have statistical significance.

### 3.4 The concept of “autonomic conflict”

In most cases, arrhythmic events induced by hERG channel blockers, torsadogenic drugs, are associated with concomitant effects on the autonomic nervous system, very similar to those observed in patients with long QT syndrome. In these patients, the triggering factors are well-known (79). These triggering factors include intense physical activities such as swimming or running, emotional stress such as sudden fear, intense excitement, or auditory stress, immersion associated with swimming, or waking up during a sleep phase, for example. These are situations in which the sympathetic nervous system is activated more or less suddenly, in the context of sometimes predominant vagal activity. Both systems are actually co-activated, at least transiently, and sometimes in a more stable manner, such as during the deceleration phase of heart rate after physical effort or during immersion swimming. This situation of immersion swimming is, in fact, one of the most favorable for the occurrence of torsades de pointes in patients with long QT syndrome (80). These situations of sympatho-vagal coactivation create what is called an autonomic conflict, where the two systems have opposing effects on heart rate. The autonomic conflict can trigger ventricular arrhythmias and torsades de pointes (81, 82), as well as atrial fibrillation (83). This situation is characterized by sequences of heart rate acceleration followed by at least one prolonged pause. In humans, cases of torsades de pointes recorded in patients who have experienced episodes of torsades de pointes are predominantly pause-dependent, meaning that a phase of acceleration followed by a pause precedes the onset of a torsades episode (84). The same pattern is observed just before episodes of torsades de pointes induced by dofetilide in cynomolgus macaques (74). As mentioned earlier, it is possible to quantify the state of sympatho-vagal coactivation using the HFAM model. This state is enhanced by a large majority of torsadogenic drugs and can become predominant, sometimes constituting up to 80% of the HF oscillations (63). Given the inverse relationship between heart rate and the QT interval, this state of sympatho-vagal coactivation is always accompanied by an increase in the amplitude of the high-frequency oscillations (HFQT) of the QT interval. This indicates a heightened sensitivity of the QT interval to changes in autonomic balance, contributing to the potential for arrhythmias like torsades de pointes. These HFQT oscillations are the primary source of beat-to-beat variability in the QT interval. They also represent the main source of short-term QT variability, which was initially visualized through Poincaré diagrams. This variability, influenced by autonomic activity, can be crucial for identifying and assessing the risk of arrhythmias such as torsades de pointes, particularly in the presence of medications that alter repolarization dynamics. These HFQT oscillations serve as an even more specific marker for torsadogenic arrhythmic risk because they are directly linked to the concept of “pause-dependence,” which precedes the triggering of torsades de pointes. High-risk torsadogenic drugs increase the amplitude of these oscillations through two mechanisms (85). The first mechanism results from the QT interval prolongation effects of these drugs. The second results from the sympathetic activation generated by the state of sympatho-vagal coactivation. Indeed, the blockage of the *β*-adrenergic component of the sympathetic component reduces the amplitude of the high-frequency oscillations of the heart rate (HFHR) and the QT interval (HFQT) induced by torsadogenic agents. The increase in the amplitude of these high-frequency oscillations clearly plays a triggering role in ventricular arrhythmias. Indeed, the suppression of high-frequency oscillations induced by the blockage of the autonomic nervous system completely eliminates the triggering of ventricular arrhythmias induced by dofetilide in healthy dogs (63). It should be emphasized that the sympathetic nervous system also plays a major role in triggering ventricular arrhythmias and torsades de pointes in patients with congenital long QT syndrome, as the first-line treatment for these patients consists of beta-blocker medications (32). In the most severe cases, treatment may involve ablation of the left stellate ganglion. The underlying question or root cause remains the mechanism that leads to this sympathetic-vagal activation, which is responsible for the increase in HF oscillations that act as the trigger. This is where the electrophysiological and hemodynamic profiles of the highest-risk torsadogenic drugs converge to adjust Coumel's triangle (Figure 2), in a context where the substrate is the prolongation of ventricular repolarization, the modulator is the autonomic nervous system, and the trigger is the sympatho-vagal coactivation and the resulting increase in HFHR and HFQT oscillations (63). As explained earlier in the hemodynamic section, drugs with *α*1-adrenergic blocking properties can provoke sympathetic-vagal coactivation through the activation of the baroreflex and other cardio-inhibitory reflex pathways in response to their arterial and venous vasodilatory effects. Several torsadogenic drugs exhibit this profile, including thioridazine, sertindole, risperidone, chlorpromazine, and droperidol. For others, the hemodynamic profile corresponds to the activation of the baroreflex, characterized by vagal bradycardia and an increase in HF oscillations in response to hemodynamic effects that produce an increase in cardiac output. This vagal bradycardia is associated with sympathetic activation in response to the transient hypotension caused by the absence of beats during the vagal bradycardia phase within the HF oscillations themselves. The examination of the pharmacological profiles of secondary or “off-target” targets indeed reveals positive inotropic properties for certain molecules, such as cisapride, known to stimulate cardiac 5-HT4 serotoninergic receptors (86). Positive inotropic effects due to the persistence of the calcium current during the plateau phase have also been reported (87) with hERG blockers causing large ventricular repolarization prolongation as a result of the blockade of the IKr current and the persistence of the late INa current. The predominance of vagal parasympathetic activity observed in patients with long QT syndrome also supports this hemodynamic hypothesis (88).

**Figure 3:**
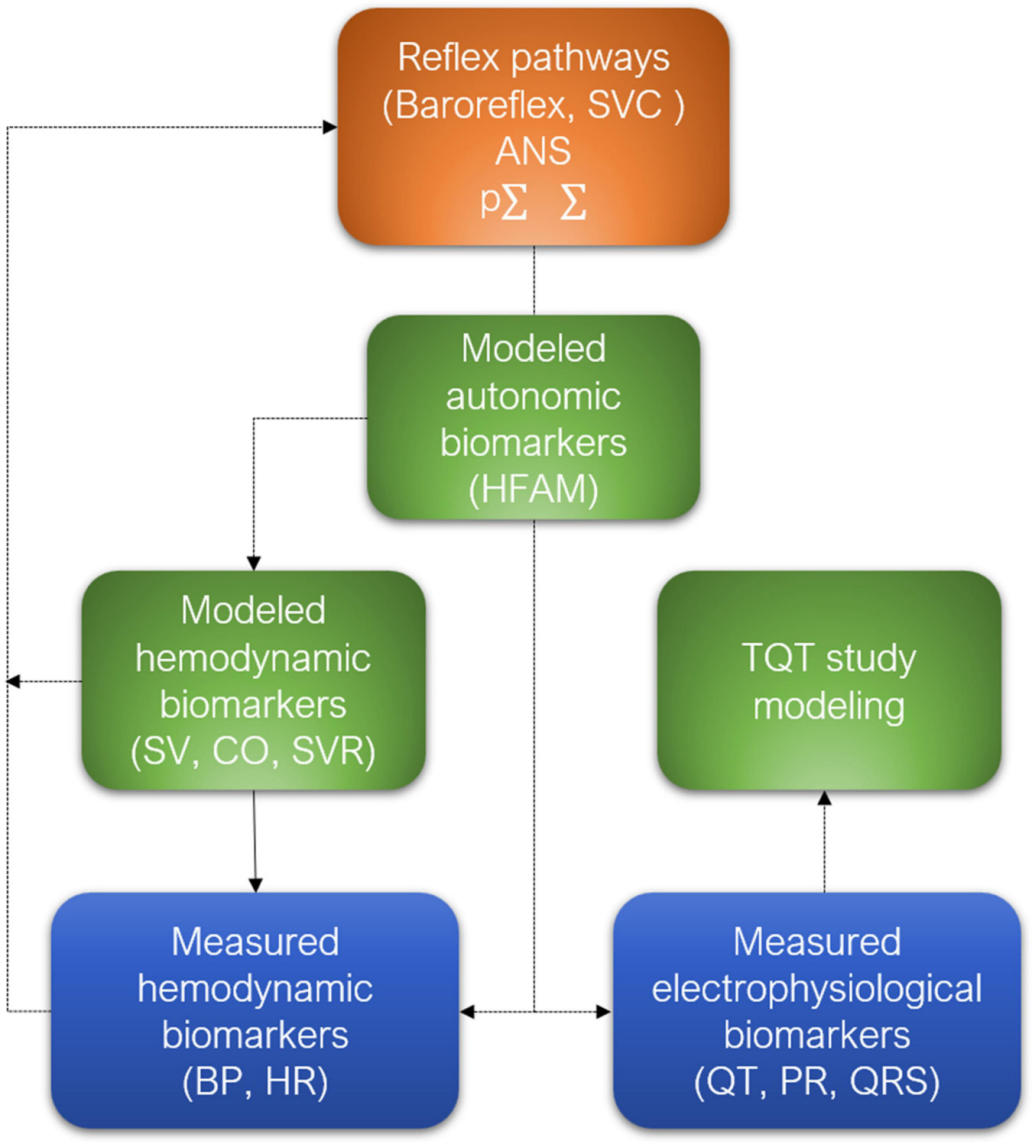
Combining biomarker measurements collected from cardiovascular safety pharmacology studies using telemetry (blue) with modeling approaches (green) of hemodynamic and autonomic biomarkers, as well as modeling data from thorough QT (TQT) clinical studies, enhances and deepens the assessment of drug-induced cardiovascular safety. When integrated into a closed-loop model incorporating reflex pathways involving the autonomic nervous system (braun), this framework also serves as a hypothesis-generating tool, enabling the identification of potential off-target effects detected during early phases of safety pharmacology.

## 4 Discussion

One of the major objectives of cardiovascular safety pharmacology is to alert clinicians about the hemodynamic and electrophysiological risks associated with new drugs being proposed to them. Without assuming the majority view of clinicians, their perception has been expressed, particularly through the need to change the paradigm regarding cardiovascular risk assessment in non-clinical studies. The main argument relies on the concept of hidden cardiotoxicity (72). This concept is based on the idea that many cardiotoxic effects observed in clinical settings only manifest in pathological contexts, and are therefore not detected in healthy models, whether animal, tissue, or cellular. These clinicians therefore urge non-clinician toxicologists and pharmacologists to revisit safety strategies and use models that are closer to the pathologies encountered in clinical settings. In a way, this concept aligns with the Coumel triangle, where the pathological context often plays a role as a substrate, such as in myocardial ischemia, heart failure, diabetes, and long QT syndromes, when it comes to torsadogenic drugs or other types of arrhythmias. Age can also be considered a substrate for hemodynamic-related cardiotoxicity phenomena, such as the risk of syncope due to reduced baroreflex sensitivity during aging (89). These unexpected cardiotoxic phenomena, associated with the pathological context, can have serious consequences and increase mortality, potentially leading to the termination of clinical trials. This was exemplified by the higher mortality observed with alpha1-blockers in clinical trials involving heart failure patients (90). In some cases, increased mortality remains unexplained, as in this example. However, the autonomic conflict induced by these pharmacological profiles may offer a plausible explanation for this population, which is particularly sensitive to such triggers, despite the drugs’ beneficial effects on cardiac preload. In this instance, the solution does not lie in a pathological model, which is inherently challenging to standardize. However, it could involve the systematic integration of markers of autonomic conflict in *in-vivo* cardiovascular safety pharmacology studies through telemetry. Some concepts take a long time to be considered as the CAST clinical studies from the late 1980s, conducted with certain antiarrhythmic drugs that inhibit the fast sodium current (“fast INa”) such as encainide or flecainide. By slowing down conduction, these antiarrhythmic drugs eliminate ventricular ectopic beats but create reentry circuits and phenomena of spatial dispersion of repolarization in myocardial infarction situations, leading to sustained ventricular arrhythmias and resulting in increased mortality in clinical trials (91). More than 25 years after these clinical studies, the strategy has significantly evolved. The Nav1.5 channel, which generates this fast sodium current, has become the second most tested channel using the patch-clamp technique, after the hERG channel, and rightfully so (92). Indeed, studying the effects on this channel helps detect drugs that may cause conduction delays. These effects on conduction result in an enlargement of the QRS complex duration, which is well correlated with patch-clamp results (93). Therefore, the term “hidden cardiotoxicity” does not seem appropriate either, as this type of risk does not require pathological models to be anticipated. The case of COX-2 inhibitors may initially seem to support the concept of hidden cardiotoxicity related to a pathological context. Several COX-2 inhibitors, such as rofecoxib and valdecoxib, were withdrawn from the market due to hypertensive and prothrombotic effects linked to the inhibition of prostacyclin (PGI2) at the level of the vascular endothelium. However, the literature does not report results obtained using the standard telemetry model on large animals used in cardiovascular safety pharmacology. The only published results on large animals were produced much later using a pentobarbital-anesthetized dog model, where an hypotensive effect with rofecoxib was reported (94). It is well known that anesthetics can interfere with and alter the cardiovascular effects of drugs. Therefore, this case appears to be more about insufficient preclinical data to adequately assess the cardiovascular safety of these drugs, rather than hidden cardiotoxicity caused by a pathological context needed to trigger a cardiotoxic effect. The detailed retrospective analysis of the reasons behind the high false positive rate (65%) between preclinical data and TQT studies also reveals, in several cases, a lack of sufficient preclinical data, which may have contributed to the decoupling (95). These findings support the hypothesis that there is significant room for improvement in safety pharmacology, particularly regarding the quality and standardization of the data produced. This hypothesis is further strengthened by retrospective analyses of literature data, which show concordance levels around 90% on the issue of QT prolongation (96). Safety pharmacologists have responded by implementing “Good Clinical Practices Q&A ICH S7B” for hERG channel and telemetry studies. However, these corrective actions may not fully meet clinicians’ expectations regarding hidden cardiotoxicity. This dissatisfaction among clinicians may be further exacerbated by the observation made by toxicologists and safety pharmacologists themselves that the “core battery” has very limited utility in terms of safety for clinical trials. It is true that it is perceived by many stakeholders in the field as a list of studies to check off (“box ticking strategy”) and, therefore, as a constraint (97). This sentiment is particularly shared by large pharmaceutical companies, which often conduct in-depth safety pharmacology investigations in a non-GLP framework before proceeding with the GLP studies of the “core battery” due to regulatory obligations. It is indeed the case that, from a 3Rs perspective, repeating these studies in a GLP mode is not optimal. On the other hand, these studies often represent the only safety pharmacology investigations that single-project companies will conduct, as they do not have the same resources and means in cardiovascular pharmacology as medium and large pharmaceutical companies. Transferring all safety pharmacology investigations conducted in the “core battery” into toxicology studies is an option that is increasingly appealing to many and is already well advanced in its process. The Irwin test is frequently replaced by the FOB (Functional Observational Battery) conducted in toxicology studies, and whole-body plethysmography is replaced by “jacket telemetry” (98). This is already the case for drugs that fall outside the scope of ICH guidelines S7A and S7B and whose cardiovascular investigations are limited to snapshot ECG recordings combined with oscillometric blood pressure measurements as part of chronic toxicology studies. Yet, the case of two messenger RNA vaccines against SARS-CoV-2 demonstrates that continuing along this path is not a viable solution. Indeed, cardiac side effects have been reported with these two vaccines, and a mechanism of action for their cardiotoxic effects has been identified (99). Specifically, in both cases, the mRNA led to intracellular calcium leakage from the sarcoplasmic reticulum due to dysfunction of the Ryr2 channel protein in the endoplasmic reticulum. These delayed effects were identified using a rat isolated cardiomyocyte model following a 72-hour incubation period to allow mRNA expression. Although the target was the same, the cardiotoxic mechanisms differed, with one case leading to cardiac arrhythmias and the other resulting in increased inotropy. This type of cardiotoxic mechanism, which impacts cardiac performance and induces arrhythmias, is fully detectable within the framework of cardiovascular safety pharmacology, both *in vivo* and *in vitro*. Furthermore, this example reinforces the value of the CiPA approach, as cardiomyocyte models are an integral part of it. Once again, this is not truly a case of hidden cardiotoxicity in the sense that the absence of a pathological context would have prevented detection of these effects. Instead, this example highlights the limitations of conventional toxicology studies and the necessity of complementing them with pharmacological approaches. This perspective aligns with the vision expressed by Zbinden in 1979 (100) who is regarded as one of the founding figures of safety pharmacology (101). Moreover, animal models used in general and cardiovascular safety pharmacology have been largely replaced by *in vitro* profiling during the early stages of drug discovery. These early safety approaches enable to target a broad range of receptors and enzymes that could potentially induce adverse effects (102, 103). Linking these huge sources of data to all other *in vitro*, in silico and *in vivo* data collected during drug development, clinical stages and pharmacovigilance is an achievable challenge, made possible by advances in artificial intelligence (104). These perspectives require cardiovascular safety pharmacology to adopt more comprehensive and mechanistic approaches, looking beyond blood pressure and arrhythmic risk as currently assessed by hERG inhibition and QT prolongation, in order to better bridge non-clinical and clinical development stages. Modeling approaches offer these insights, as illustrated by the combination of biomarker measurements from cardiovascular safety pharmacology studies using telemetry with modeling approaches of hemodynamic and autonomic biomarkers, together with modeling of thorough QT (TQT) clinical studies (Figure 3).
